# How can we exploit above–belowground interactions to assist in addressing the challenges of food security?

**DOI:** 10.3389/fpls.2013.00432

**Published:** 2013-10-30

**Authors:** Peter Orrell, Alison E. Bennett

**Affiliations:** Ecological Sciences, James Hutton InstituteInvergowrie, Dundee, UK

**Keywords:** arbuscular mycorrhizal fungi, rhizobia, PGPR, pollination, herbivory, intercropping, indirect defense, predators

## Abstract

Can above–belowground interactions help address issues of food security? We address this question in this manuscript, and review the intersection of above–belowground interactions and food security. We propose that above–belowground interactions could address two strategies identified by Godfray etal. (2010): reducing the Yield Gap, and Increasing Production Limits. In particular, to minimize the difference between potential and realized production (The Yield Gap) above–belowground interactions could be manipulated to reduce losses to pests and increase crop growth (and therefore yields). To Increase Production Limits we propose two mechanisms: utilizing intercropping (which uses multiple aspects of above–belowground interactions) and breeding for traits that promote beneficial above–belowground interactions, as well as breeding mutualistic organisms to improve their provided benefit. As a result, if they are managed correctly, there is great potential for above–belowground interactions to contribute to food security.

## INTRODUCTION

The number of recent reviews (Wolters et al., 2000; van der Putten et al., 2001; Bardgett and Wardle, 2003; Porazinska et al., 2003; van Dam et al., 2003; Wardle et al., 2004; Bardgett et al., 2005; Bezemer and van Dam, 2005; van der Putten et al., 2009; van Dam and Heil, 2011; Soler et al., 2012) as well as this special issue have highlighted the importance of above–belowground interactions in both natural and agricultural systems. Throughout this paper we will refer to above–belowground interactions as any influence of a belowground process on an aboveground process and vice versa in the vein of the recent book by Bardgett and Wardle (2010), as it is changes in these processes that broadly influence an agricultural ecological network. In particular, we will focus on how different below- and above-ground organisms alter these processes. Above–belowground research has highlighted the influence of belowground organisms on plant defense against herbivory (Dean et al., 2009; Gehring and Bennett, 2009; Koricheva et al., 2009; Pineda et al., 2010; Thamer et al., 2011; Zamioudis and Pieterse, 2012) and pathogens (reviewed in Borowicz, 2001; Hayat et al., 2010; Beneduzi et al., 2012; Bhattacharyya and Jha, 2012; Zamioudis and Pieterse, 2012), as well as indirect defense (Gange et al., 2003; Guerrieri et al., 2004; Rapparini et al., 2008; Fontana et al., 2009; Hempel et al., 2009; Leitner et al., 2010; Hoffmann et al., 2011; Wooley and Paine, 2011; Schausberger et al., 2012), and the role of aboveground herbivory on nutrient cycling (Hunter, 2001). These factors (herbivore defense, pathogen defense, and access to nutrients) are major concerns in agriculture, and increasing all three in a sustainable manner are goals of food security.

In their seminal review Godfray and colleagues identified three challenges to future food security: providing desired food to a larger richer population, providing the aforementioned food in a sustainable manner, and abolishing hunger worldwide. Here they identified five strategies for meeting those challenges: “closing the yield gap,” “increasing production limits,” “reducing waste,” “changing diets,” and “expanding aquaculture” (Godfray et al., 2010). Employing these strategies will become increasingly difficult as the availability of chemical inputs such as fertilizers, pesticides, and fungicides, declines due to increased costs and reduced access to materials such as fossil fuels (Woods et al., 2010) and phosphorus (Cordell et al., 2009), as well as changing legislation (Birch et al., 2011). As a result, we propose that in combination with changes in social, cultural, political, technological, and scientific aspects of agriculture, managing above–belowground interactions creates an opportunity to assist in addressing two of the food security strategies above – closing the yield gap, and increasing production limits (**Table 1**) in a sustainable manner. Although above–belowground interactions cannot address these issues in their entirety, they hold the potential to form an important constituent in addressing food security. In particular, certain above–belowground interactions can be exploited to improve plant growth and nutrition, benefits from mutualists, and defense against antagonists. We explicitly describe below how these can influence the above food security strategies.

**Table 1:** Table listing the various mechanisms that could be used to address the two food security strategies addressed in this manuscript, and the potential contributions above–belowground interactions could make to these mechanisms.

Food security strategy	Mechanism	Above–belowground contribution
Reducing the yield gap	Increase crop growth	Benefits from priority effects
		Improved mutualistic interactions
	Reduce losses to pests in field	Enhanced plant defense to pests
		Reduction in negative interactions
Increasing production limits	Intercropping	Overyielding
		Benefits from niche dynamics
		Facilitation
		Enhanced plant defense to pests
	Breeding	Improved beneficial above–belowground interactions
		Improved mutualistic organisms

## CLOSING THE YIELD GAP

A yield gap is the difference between potential and realized harvests in agriculture, with losses resulting from inadequate capacity to access and utilize water, nutrients, appropriate seed stock, and knowledge, as well as inadequate management of pests, pathogens, soil conditions, and biodiversity (Godfray et al., 2010). In low-income areas, investment in closing this gap typically does not match returns on other uses of capital and labor, creating a situation where it is economically sub-optimal to raise production to maximum potential yield (Godfray et al., 2010). Combined with peak phosphorus (Cordell et al., 2009) and increases in the price of fossil fuels (Woods et al., 2010), the costs of intensive farming techniques are rising. Exploiting beneficial above–belowground interactions, however, could form a part of a more cost effective, sustainable, long-term strategy to aid in closing yield gaps (**Table 1**). Particularly, in the context of our review, these advantages could be achieved through the promotion of beneficial relationships between crops and mutualistic organisms, and the mitigation of crop losses resulting from negative interactions with pests and pathogens, which can include both direct losses from pest damage, and indirect losses such as changes in the way in which the plant interacts with other species. Crop losses due to pathogens have been estimated to account for between 9 and 21% of global losses (Oerke, 2006), and losses from herbivores have been shown to significantly decrease yields (Strauss and Murch, 2004). Typically, pathogens are controlled through the use of chemical applications, though rising costs, changes in legislation (Birch et al., 2011), and cultural shifts toward sustainable farming have led us to seek alternative solutions. Research has shown the potential to mitigate stresses from pests and pathogens when a host plant interacts with other pests (reviewed in van Dam and Heil, 2011; Soler et al., 2012), mutualists (Gehring and Bennett, 2009; Pineda et al., 2010), and through the utilization of priority effects with these organisms. Here we will outline how above–belowground interactions may be used within a toolset that incorporates social and technological changes in agriculture to mitigate yield gaps, and realize harvests closer to their maximum potential (**Table 1**).

## PROMOTING BENEFICIAL MUTUALISMS

Beneficial interactions between crop species and mutualists could allow us to reduce yield losses from both biotic and abiotic above–belowground stresses. Along with the supplemental addition of mutualists, the promotion of conditions that are favorable to mutualists could increase the benefits of these relationships, which hold the potential to offer many of the services currently provided by synthetic nutrients and agro-chemicals. Here we provide a sample of some of the above–belowground interactions influenced by mutualisms that could be utilized in agriculture.

### AM FUNGI

As well as supporting the plant through their direct benefits on plant growth and yield in commercial crops (Ceballos et al., 2013), arbuscular mycorrhizal (AM) fungi can also indirectly benefit plants by reducing herbivore visitation rates, as well as reducing the impact of herbivore damage (reviewed in Gehring and Bennett, 2009). AM fungi have the potential to improve defense against pests and pathogens through different means. Firstly, infection by AM fungi appears to promote direct plant defenses (such as induced secondary defensive chemicals), defending plants against both generalist chewing herbivores (reviewed in Gehring and Bennett, 2009; Koricheva et al., 2009), and reducing the impacts of pathogens (reviewed in Borowicz, 2001). Secondly, AM fungi can promote indirect defenses, such as the release of volatiles that attract herbivore enemies to defend against specialist and sucking herbivores (Gange et al., 2003; Guerrieri et al., 2004; Rapparini et al., 2008; Fontana et al., 2009; Hempel et al., 2009; Leitner et al., 2010; Hoffmann et al., 2011; Wooley and Paine, 2011; Schausberger et al., 2012). Typically, commercial applications of AM fungi are used exclusively to promote the production of biomass; however the studies above suggest they can prevent losses in yields due to herbivory. These effects, however, can be AM fungal species specific, and isolation of the most appropriate inoculum is required (Gange et al., 2003, 2005; Bennett et al., 2009).

Arbuscular mycorrhizal fungi have been shown to be able to affect both above and belowground pathogens, altering disease resistance in crops (reviewed in Borowicz, 2001; Dakora, 2003). The initial colonization of a plant by AM fungi activates induced systemic responses, which can leave crops in a primed state, better able to defend themselves against subsequent attacks from pathogens (Van der Ent et al., 2009; Jung et al., 2012). Changes caused by AM fungi in plant defensive compounds (Van der Ent et al., 2009; Jung et al., 2012), gene expression (Hause et al., 2002; Strack et al., 2003), above and belowground plant architecture (Azcón-Aguilar and Barea, 1997; Whipps, 2004; Vierheilig et al., 2008), and competition for resources (Azcón-Aguilar and Barea, 1997; Whipps, 2004; Vierheilig et al., 2008) can reduce the efficiency of pathogenic infections, and potentially decrease losses incurred from fungal and viral attacks (Fusconi et al., 1999; Slezack et al., 1999). AM fungal induced changes in the quantity, quality, and partitioning of JA (Van der Ent et al., 2009; Jung et al., 2012), ethylene (Van der Ent et al., 2009), and phenolic compounds (Yao et al., 2007) alter a plant’s defensive capabilities, and can be comparable in effectiveness to the use of agro-chemicals. For example, a study that compares the effects of AM fungi and fungicide on the control of root rot (*Rhizoctonia solani*) in *Phaseolus vulgaris,* Abdel-Fattah et al. (2011), found no significant difference between the control of *R. solani* in terms of yield from both fungicide (6.7 g/plant) and AM fungi (6.9 g/plant). A study by Hu et al. (2010), found that inoculation with a community of AM fungi decreased the impact of *Fusarium oxysporum *f. sp. *cucumerinum *in* Cucumis sativus *L. and successfully improved fruit yields equal to those in the control treatment, unlike inoculation with a single AM fungal species. AM fungi are not always effective against plant pathogens, but there is potential for AM fungi to play a role in pathogen protection (Gernns et al., 2001). Biotic factors such as the species of AM fungi and pathogen (Azcón-Aguilar et al., 2002; Vierheilig et al., 2008; Hu et al., 2010; Wehner et al., 2010), and abiotic factors such as temperature, soil moisture, and soil P concentration (Singh et al., 2000; Vierheilig et al., 2008) play an important role in determining the direction and efficiency of this relationship. The outcome of AM fungal-pathogen interactions also often depends on the class of the pathogen, though overall AM fungi tend to decrease damage from fungal pathogens (Borowicz, 2001).

With the advent of AM fungal seed coatings, there is now the possibility to create tailored AM fungal seed coatings, specific for an individual variety of crop species and its expected stresses. With commercial use of AM fungi increasing in agriculture and current socio-economic shifts toward sustainable farming, it is likely that AM fungal coated seeds will become more prevalent within agriculture. To successfully integrate AM fungal interactions, however, we will need to change agricultural management. In areas of intensive agriculture and high nutrient input, we often see a reduction in indigenous AM fungi (reviewed in Gosling et al., 2006; Fester and Sawers, 2011). The application of fungicidal chemicals often treats a broad range of organisms, providing control of detrimental pathogens, but negatively influences the abundance of beneficial microbes such as AM fungi and fungal endophytes (Harvey et al., 1982; Schreiner and Bethlenfalvay, 1997; Kjøller and Rosendahl, 2000). Trade-offs may also be present, and benefits in the field may not match those observed in the glasshouse. For example, plants inoculated with AM fungi may increase the growth of nematodes, thereby increasing the potential for negative effects from pathogens (Borowicz, 2001). Thus, more research is needed to fully understand these above–belowground interactions, and identify their potential to combat in field losses caused by pathogens whilst also increasing yield. Thus, maintaining indigenous mutualists and utilizing inocula to take advantage of the wide range of interactions provided by AM fungi, in combination with social, economic, political, and technological changes to agriculture, may form an important role in assisting efforts to mitigate current and future yield gaps.

### PLANT GROWTH PROMOTING RHIZOBACTERIA

First defined by Kloepper and Schroth (1978), plant growth promoting rhizobacteria (PGPR) are free-living and endophytic rhizosphere bacteria that are able to influence plants directly through the promotion of plant growth, and indirectly through the mitigation of plant stresses. Studies have shown that PGPR can increase plant resistance to herbivores and pathogens through induced systemic resistance (ISR) generated from changes in jasmonic acid (JA), salicylic acid (SA), ethylene, and other metabolites (Van der Ent et al., 2009). PGPR (and other soil mutualists) do not tend to enhance biosynthesis of these hormones or gene expression, but rather appear to prime host plants for attack by initiating the salicylic acid pathway in a host plant, whilst preventing the completion of all products in the pathway, thereby leaving the plant able to respond more rapidly to attack (reviewed in Pozo and Azcon-Aguilar, 2007; Van der Ent et al., 2009; Pineda et al., 2010; Jung et al., 2012; Zamioudis and Pieterse, 2012). PGPR can be cultured under laboratory conditions, and thus can readily be added as a soil amendment. The use of tailored rhizobia seed coatings to promote crop yields already occurs, but there is also the potential to coat seed with rhizobia (and even PGPR) that increase plant defense or tolerance. Despite the potential that PGPR offer, research has found it difficult to translate results observed in the greenhouse to real-world field trials. Thus, with more research into their practical application, selecting inocula to take advantage of beneficial interactions could form an important role in developing a package of solutions to help reduce global gaps in yields. More research is needed on the activity of these bacteria, but PGPR present an excellent opportunity for promoting plant defense in agricultural systems.

### EARTHWORMS

Earthworms also have the potential to influence crop yields via above–belowground interactions. Earthworms have a primary role as decomposers and can influence plant–soil interactions through the release of nutrients and changes in soil structure (Scheu, 2003). Earthworm activity can have two impacts on above–belowground interactions that promote crop yields: first, earthworms and their activity can reduce herbivory in host plants, and second, earthworms can increase the abundance of other soil organisms that reduce herbivory in host plants. Earthworms can positively and negatively influence damage by above ground herbivores through changes in primary and secondary metabolites, as well as the expression of stress responsive genes (Wurst et al., 2004a,b; Poveda et al., 2005; Ke and Scheu, 2008; Wurst, 2010), and therefore increase plant tolerance to herbivory (Blouin et al., 2005). When combined with their ability to improve plant nutrition, this could lead to mitigation against losses in yields from herbivory. Additionally, earthworms can alter rhizosphere microbial communities in ways that benefit crop plants. For example as microorganisms pass through an earthworm’s gut microbial community structure is altered. The abundance of PGPRs can increase (Wurst, 2010) thereby priming plants for defense against above and belowground pest and pathogen attack. There are two possible ways to improve earthworm abundance in agricultural fields, and thereby take advantage of both their growth and plant defense-enhancing properties. (1) The addition of organic material to promote the abundance of earthworms (Hole et al., 2005; Marhan and Scheu, 2005), and (2) the addition of supplemental native beneficial earthworms. Earthworms provide the potential for a relatively low cost sustainable option not only to mitigate herbivory, but also to improve soils, and in turn overall plant quality thereby reducing yield gaps created by both pests and lack of nutrients. Vermicompost (worm worked soil) has also been found to be effective against herbivores, even without worms present (Edwards et al., 2004). Earthworm populations are typically already found in most agricultural systems, however managing agricultural land to promote their abundance and effectiveness as described above could play a role in mitigating yield gaps and reducing in field losses from pests. For example, NPK fertilization rather than the addition of farmyard manures has been illustrated to reduce the biomass of some earthworms (Marhan and Scheu, 2005). Thus, worms and vermicompost promote beneficial interactions and in combination with changes in cultural and technological approaches to agriculture have the potential to assist in closing the yield gap and reducing in field losses without reliance on intensive agro-chemicals.

### ENHANCING POLLINATION SERVICES

With increased pollination, herbivore-damaged plants can at times produce equal yield to undamaged plants (Strauss and Murch, 2004). As a result, improving pollination services may be more effective both in terms of yield and cost than combating pests themselves. Often due to changes in reproductive plant characteristics, studies have documented increases in pollination rates in plants associated with AM fungi (Gange and Smith, 2005; Wolfe et al., 2005). Pollinators typically have been managed through aboveground mechanisms, however AM fungi can influence plant size (Smith and Read, 2008), inflorescence size (Gange and Smith, 2005; Varga and Kytoviita, 2010) and number (Gange and Smith, 2005), quality of pollen and nectar (Gange and Smith, 2005; Varga and Kytoviita, 2010), and timing of reproduction (Koide, 2000). Due to changes in these plant characteristics studies have documented increases in pollination rates in plants associated with AM fungi (Gange and Smith, 2005; Wolfe et al., 2005). In one study, AM fungi enhanced some reproductive traits and yields similar to that when fertilized, although genotypes and individual traits responded differently (Poulton et al., 2002). If plant traits such as these can be enhanced by belowground mutualists, then benefits may be observed aboveground in terms of yield through increased pollination. The ability to sustainably enhance both plant nutrition and pollination efficiency may play an important role in assisting to close yield gaps, and curtail current and future global food shortages if used in combination with social and technological changes in agriculture. This is a growing area of knowledge, and more research is needed into crop plant responses to AM fungi in order to specifically target AM inocula to enhance pollination rates, and in turn production, thereby assisting in reducing yield gaps.

## MITIGATING LOSSES FROM NEGATIVE INTERACTIONS

### PRIMING PLANT DEFENSES

One means of reducing the yield gap is to improve plant defense against herbivores that damage crops. As discussed above, mutualistic belowground organisms (such as AM fungi, rhizobia, earthworms, and PGPR) can enhance plant defense against herbivory (Dean et al., 2009; Gehring and Bennett, 2009; Koricheva et al., 2009; Pineda et al., 2010; Thamer et al., 2011). While these mutualisms often increase plant nutrition thereby allowing plants to allocate more resources to defense (Bennett et al., 2006; Kempel et al., 2010), the antagonistic action of these mutualistic organisms against pests appears to be due to priming of plant defenses such as JA, salicylic acid, ethylene, and other metabolites. Although we are still unsure of the extent of priming (Karban, 2011), using priming in agriculture to enhance induced resistance to pests is a growing area of interest (Ahmad et al., 2010; Hayat et al., 2010; Conrath, 2011; Walters et al., 2013). More research is needed in this area to determine the efficacy of this approach in agriculture (Walters et al., 2013), but if priming capacity is widespread in crop plants then the utilization of soil mutualistic microbes to enhance plant defense against herbivores and other pests via priming will aid in closing the yield gap by reducing loss to pests within agricultural production.

### HARNESSING ADVANTAGES FROM PRIORITY EFFECTS

Priority effects are the interactions observed in plants in response to an initial invader, and can change plant physiology, affecting subsequent species (Strauss, 1991). Research into above–belowground interactions has revealed that these effects can play a relatively large role in determining the defensive state achieved by a plant from its relationship with mutualists and parasites (e.g., Gerber et al., 2007). The first herbivore to arrive on a host plant changes plant chemistry thereby influencing the entire community of visiting insects throughout the remainder of the growing season (Chase et al., 2000; Gerber et al., 2007; Soler et al., 2007a; Poelman et al., 2010). Herbivore priority effects however, are broad, and also influence plant architecture and a range of other factors, thereby indirectly influencing pollination and in turn overall yield (e.g., Gerber et al., 2007). As the success and efficiency of pollination is often a large determinant of yields, priority effects that promote increased pollination may outweigh the negative impact of the primary herbivore, leading to an indirect mitigation of yield loss. Thus, influencing the timing of arrival of an above or belowground herbivore could be used to prime plants against more serious attacks later in the season and promote increased pollination. Crops could, for example, be treated with a low impact herbivore that causes minimal yield losses, invoking induction, or priming of secondary chemicals in the plant for more serious attacks later. Herbivory, however, is not the only plant interaction that experiences priority effects, and these effects can determine the benefits provided by mutualists. The initial AM fungus that colonizes a host plant root appears to dominate the root system at least at the beginning of the growing season (Hepper et al., 1988; Dumbrell et al., 2011), with rapid colonization giving it a competitive edge potentially inhibiting colonization by other AM fungi (Schwartz et al., 2006) and subsequent attacks by herbivores (reviewed in Pineda et al., 2010; Zamioudis and Pieterse, 2012). Priority effects are often observed within crops grown in agriculture, and taking advantage of these effects, especially when attempting to augment farming practices to promote beneficial above–belowground interactions, could further enhance sustainable efforts to close yield gaps and reduce loss to pests.

### VOLATILES AND SIGNALING

There are a growing number of studies demonstrating that belowground interactions can influence aboveground predator and parasitoid recruitment to host plants, and thus maximizing the influence of these belowground interactions should reduce herbivore abundance and increase crop yields by reducing in field losses from pests (Shrivastava et al., 2010). Several studies have demonstrated that AM fungi can alter volatile release and/or enhance predator attraction to host plants (Gange et al., 2003; Guerrieri et al., 2004; Rapparini et al., 2008; Fontana et al., 2009; Hempel et al., 2009; Leitner et al., 2010; Hoffmann et al., 2011; Wooley and Paine, 2011; Schausberger et al., 2012). For example, Guerrieri et al. (2004) showed that AM fungi increased volatile release in tomato (*Lycopersicon esculentum* Miller), and Schausberger et al. (2012) demonstrated that AM fungi changed the composition of released volatiles, resulting in increased attraction of a predator to the herbivore feeding on the common bean plant (*P. vulgaris*). The effects of AM fungal enhanced predator attraction are largely untested in crop fields, presenting a research opportunity to explore AM fungal influence in field environments. In the only field test of AM fungal influence on parasitoid attraction, results showed that parasitism was lower on plants where AM fungi had not been suppressed with fungicide (Gange et al., 2003). PGPR have also been credited with increasing parasitoid attraction to field plots treated with PGPR (Saravanakumar et al., 2008), although results are not always consistent (Boutard-Hunt et al., 2009). As a result, although more research needs to be conducted, belowground mutualists show great promise as promoters of crop plant indirect defense.

Microbes, however, are not the only organisms to influence plant volatiles and attraction of parasitoids to herbivores – herbivores themselves can also influence these characteristics. Many studies examining the influence of root herbivores on aboveground parasitoid attraction have revealed that root herbivores decrease attraction and performance of parasitoids on shared host plants possibly due to increased chemical defenses of plants (reviewed in Soler et al., 2012). This is not always the case, as when *Brassica nigra* was subjected to root herbivory by *Delia radicum* it led to increased parasitoid attraction to herbivores on aboveground shoots of the host plant as well as neighboring host plants (Soler et al., 2007b). As a result, managing root herbivory could have consequences for plant indirect defense, although the consequences are likely to be system specific. Similarly, aboveground interactions can also influence predator attraction belowground. For example, aboveground herbivory by *Pieris brassicae* on *B. nigra* reduced the mass of both root herbivores and their parasitoid, likely due to changes in plant chemistry (Soler et al., 2007a). Fewer studies have been conducted on this type of interaction, but there is the potential to manage root herbivores utilizing above-ground interactions if we gain more knowledge about their influence belowground. Thus, managing herbivores on one side of the ground horizon is likely to have impacts on the other side, and if we can harness further knowledge of these interactions there is potential to apply this research to increase plant defensive states and better protect against losses from pests.

### EXTRAFLORAL NECTAR

Extrafloral nectar (EFN) glands (extrafloral nectaries) produce supplemental nectar in various plant organs outside of the flower for the recruitment of beneficial predators and top-down control of herbivores. Studies have shown them to occur in 1–2% of vascular plant species, including crops such as Pumpkins, Squashes, and Gourds (*Cucurbita *spp*.*), Cassava (*Manihot esculenta*), Broad Bean (*Vicia faba*), Cow Pea (*Vigna unguiculata)*, Lima Bean (*Phaseolus lunatus*), Sunflower (*Helianthus annuus*), and Sesame (*Sesamum indicum)*, amongst others (Weber and Keeler, 2013). Here we will discuss how belowground influences from both pests and mutualists are able to alter aboveground top-down control through influencing EFN production. EFN production can be induced by attack from pests, and is thought to be regulated by JA (Heil, 2008). Wäckers and Bezemer (2003), for example, found that root herbivory by wireworms (*Agriotes lineatus*) and mechanical root damage both increased EFN production in cotton plants (*Gossypium herbaceum*). AM fungi have been shown to both positively and negatively affect the abundance of EF nectaries and EF nectar production (Laird and Addicott, 2007). Thus, there is potential in a number of crop species for belowground organisms to influence plant defense both positively and negatively via their influence on extra floral nectaries.

Extrafloral nectar also influence pollination. One study found that foliar nectar production was induced in response to fruit damage, whereas bracteal nectar was found to be higher in times of pollination, and decreased under herbivory (Wäckers and Bonifay, 2004). If belowground interactions are able to influence EFN then it is likely that we will see changes in both herbivore control and pollination, resulting in changes in yield from both a reduction in field losses from pests and increased production. Promotion of EFN provides a novel solution to sustainable crop protection, although to take advantage of these induced defenses, we must understand how a range of biotic and abiotic factors and interacting species affect EFN production, as well as their effects across trophic levels. Additionally, the number of crop species producing EFN is limited, and as such will not be applicable to all agricultural systems. Despite this, there is potential to utilize EFN to mitigate losses caused by herbivory if used in conjunction with other amendments to agricultural practice.

## INCREASING PRODUCTION LIMITS

Godfray et al. (2010) argue that obtaining food security by increasing production limits predominantly depends upon changes in plant breeding and the acceptance of GM crops. We propose, in addition, that changes in farming practices (to incorporate intercropping) and inoculation of soils with beneficial microbes could equally contribute to increased production limits in crops (**Table 1**).

### INTERCROPPING

As population levels rise available suitable land for agriculture will decline (Godfray et al., 2010), and agriculture will need to find ways to gain more food from less space. An increasingly common way to increase yields with less land is intercropping (reviewed in Knoerzer et al., 2009; Palaniappan et al., 2009) – a technique that takes advantage of above–belowground interactions. Intercropping has been around since the dawn of agriculture (reviewed in Knoerzer et al., 2009; Palaniappan et al., 2009), and farmers who practice intercropping have long known that certain combinations of plants produce the greatest yield. Researchers have shown that the right combination of crops can actually lead to overyielding (greater yield in intercropping than in monoculture; Francis, 1989; Vandermeer, 1992), and above–belowground interactions and niche dynamics play a big role in overyielding. Intercropping has the potential to influence two types of above–belowground interactions. Firstly, as detailed above, belowground organisms such as AM fungi and root herbivores, etc. have the potential to affect aboveground organisms such as herbivores and pollinators and vice versa. Secondly, belowground processes influenced by intercropping such as nutrient cycling can influence aboveground plant productivity and communities, producing a cascading effect of influence across the agricultural ecological network (Bardgett and Wardle, 2010). Across the world, farmers have known for countless generations that growing legumes and cereals together increases the N nutrition of cereals leading to overyielding of cereals, particularly in unfertilized fields (e.g., Hauggaard-Nielsen and Jensen, 2005). However, in many intercropping combinations nitrogen fixation plays little or no part in the process of overyielding. Instead, facilitation, niche partitioning, and improved pest control are credited with improving yields. There are several examples of facilitation in which both legume and non-legume plant species aid uptake of limiting nutrients and water in neighboring species (reviewed in Hauggaard-Nielsen and Jensen, 2005). For example, the root exudates of legumes frequently release bound P (Hinsinger, 2001; Li et al., 2007), maize can improve the nutrition of plants with high iron needs (Kamal et al., 2000; Zuo et al., 2000), and growth of maize and pea together leads to increased water uptake for pea (Mao et al., 2012). Intercropping yields can also benefit from niche partitioning in which crop species utilize different soil and aboveground resources spatially and temporally leading to better resource (nutrient, light, water) utilization than when grown in monoculture (reviewed in Zhang et al., 2010). Finally, intercropping has been shown to reduce pest (both microbial and arthropod) attack (Zhu et al., 2000; Ndakidemi, 2006; Letourneau et al., 2011; Ratnadass et al., 2012). The pest control benefits of intercropping can be due to spatial and temporal variation introduced by interactions among species, allelopathy, soil suppression of pests, improved resistance due to interactions among species, natural enemy promotion and conservation due to improved/more diverse resources, and increased vegetation complexity (reviewed in Letourneau et al., 2011; Ratnadass et al., 2012). The use of intercropping fosters the development of a community of crop plants and other organisms to produce an environment that promotes beneficial interactions, with benefits in yield at times resulting from beneficial above–belowground interactions, as well as a range of biotic and abiotic factors. Intercropping, however, is not always beneficial, and as reviewed in Letourneau et al. (2011), does not always increase yields. Similarly, inefficiencies in other areas of the production system, such as a reduction in harvest efficiency may outweigh the benefits provided in large scale commercial operations. Despite this, with continued research intercropping has the potential to increase production limits within some agricultural systems.

### PHENO/GENOTYPES, AND PLANT BREEDING

Modern agriculture has long made use of phenotypic and genotypic variation in plant growth and plant responses to pests to increase production limits. If we are to utilize above–belowground interactions within agriculture, we must develop varieties of crops that are best able to take advantage of beneficial above–belowground interactions, as well as breeding beneficial organisms and harnessing appropriate pheno/genotypes to provide the maximum benefit to the plant. To date, the majority of plant breeding has been targeted to increase yields, and improve factors such as taste, appearance, and tolerance to biotic and abiotic factors such as diseases and environmental conditions. Here we propose that crop breeding take advantage of a wider pool of genotypic and phenotypic variation, and (1) breed plants for greater responses to above- and belowground organisms in combination, and (2) breed above- and belowground non-plant organisms that interact with crop plants. There are several different directions these types of breeding programs should, and in some cases already are, taking.

First, breed crop plants themselves to (a) best address typical above–belowground interactions, (b) respond to the most likely attacker in an environment, and (c) use above- or belowground mutualists to best promote crop yields or plant defense. Breeding crop plants to best address typical above–belowground interactions could be likened to breeding the best crop ideotype for all conditions, in particular, a variety best able to resist pathogen and herbivore attack and take advantage of mutualists (such as rhizobia, AM fungi, or foliar endophytes) thus promoting crop yields. Producing a best all round crop plant has long been the goal of crop breeding, but the emphasis in this effort has predominantly been focused on breeding for abiotic factors (such as drought tolerance) and pest defense. The ability to breed for the best all round plant will depend on whether there are trade-offs between different crop characteristics. For example, four genotypes of *Senecio jacobaea* were found to allocate pyrrolizidine alkaloids differently according to damage either above or below ground. Shoot herbivory was found to decrease concentrations of senecionine in the roots, suggesting that there may be a trade-off between defense allocation to shoot or root (Hol et al., 2004). Other such potential trade-offs have been identified. For example, variation in AM fungal colonization is negatively correlated with fungal endophyte leaf colonization (Mack and Rudgers, 2008). While the mechanism behind many of these trade-offs is still not well understood, there are other trade-offs for which we have significant knowledge, specifically the trade-offs between the jasmonic and salicylic acid pathways. This trade-off has been documented to influence the outcome of above–belowground interactions. The JA pathway tends to be activated in response to necrotrophic pathogens and chewing herbivores, and the salicyclic acid (SA) pathway tends to be activated in response to biotrophic pathogens and piercing or sucking insect herbivores (reviewed in Thaler et al., 2012). Increasing evidence suggests that when a plant is attacked by enemies with different strategies, a plant can rarely defend against both strategies simultaneously (reviewed in Stout et al., 2006; Thaler et al., 2012; Hauser et al., 2013). However, the trade-off between the JA and SA pathways does not just influence interactions between above- and belowground antagonists. For example, AM fungi and rhizobia trigger the signal cascade of the salicylic acid pathway when they colonize host plants, but then turn this cascade “off” – a process which appears to prime plants for defense against attack by future enemies (Pozo and Azcon-Aguilar, 2007; Jung et al., 2012). However, this priming appears to be most effective against chewing generalist insect herbivores as opposed to “sucking” specialist herbivores (Koricheva et al., 2009). As a result, breeding for a variety that performs well under all circumstances may be limited by intrinsic trade-offs in defense allocation or even allocation to mutualists.

Instead, it may make more sense to breed for traits that promote specific above–belowground interactions. Climatic conditions can influence the prevalence of pests and even mutualists. For example, *Myzus persicae*, which can rarely survive cold (consistently below 2°C) winter conditions frequently prevalent in northern latitudes, tend to migrate northward limiting their abundance at many northern latitude sites until late in the growing season (van Emden et al., 1969) when they are less likely to impact crop yields. As a result, breeding for defense against *M. persicae* in northern latitudes makes little sense – especially if there is an intrinsic trade-off between the ability to defend against aphids and other more common herbivores in that environment. Site history can also influence the likelihood of specific above–belowground interactions. Previous potato growth in a field can increase the prevalence of root knot nematodes (reviewed in Trudgill et al., 2003), thereby increasing the need to plant varieties with greater resistance to root knot nematode. Site history can also influence the prevalence of other partners – for example, soil disturbance such as tilling can reduce the diversity and abundance of AM fungi (Fester and Sawers, 2011; Bennett et al., 2013a), which could have significant consequences in some of the scenarios above. For example, AM fungi have been shown to enhance belowground induced defense responses to the root herbivore vine weevil (Bennett et al., 2013b), so limiting the presence of AM fungi by tilling could increase the susceptibility of crop plants to vine weevil in the field. Thus there are several cases where breeding crops for environmentally specific above–belowground interactions could increase production limits.

As mentioned above, traditional breeding programs have focused on breeding for tolerance to abiotic and biotic stresses; however, in the future, it may be wiser to breed plants capable of taking advantage of above- and belowground mutualists. With the decline of fossil fuels (Woods et al., 2010) as well as phosphorus (Cordell et al., 2009) available for the production of fertilizers and other chemical inputs, maintaining food security in the future will rely heavily on non-chemical means of nutrient supply and pest defense. Agriculture is already utilizing the legume–rhizobia mutualism to improve nitrogen nutrition, and research suggests that rhizobia can also improve plant defense against herbivores (Dean et al., 2009; Thamer et al., 2011). As a result, utilizing mutualisms such as rhizobia, AM fungi, and endophytes could improve plant nutrition and defense against enemies. However, research suggests that breeding practices have selected against associations with the below-ground mutualists AM fungi and rhizobia (Bennett et al., 2013a; but see Lehmann et al., 2012). Modern breeding has often occurred under optimal nutrient conditions thereby making nutritional mutualists redundant, and under high soil disturbance which reduces the prevalence of soil mutualists (Fester and Sawers, 2011; Bennett et al., 2013a). Some countries have already taken this strategy on board by breeding for increased response to rhizobia in legumes thereby increasing nitrogen uptake (e.g., Brazil; Alves et al., 2003). This type of breeding can occur in non-leguminous species as well. For example, it has been proposed that low N applications have selected for sugarcane (*Saccharum* spp.) that gain the bulk of their N requirement from free-living nitrogen fixing diazotrophs in the soil (Taulé et al., 2012; Urquiaga et al., 2012). As a result, breeding of crop plants for response to mutualists will be required to take advantage of nutrient uptake and plant defense potential.

The vast majority of breeding programs for improved food security have focused on breeding the crop plants themselves; however, we propose a second breeding strategy: breed above- and/or belowground organisms to promote crop yields. This strategy is likely to be more effective for symbiotic interactions than other types of above–belowground interactions, and there are a wide variety of symbiotic interactions of which to take advantage. Root mutualists, AM fungi, and rhizobia, are already being applied as seed coatings to agricultural species. This is a well-established practice for rhizobia, but a relatively new practice for AM fungi. There are priority effects in colonization of root systems (Hepper et al., 1988; Dumbrell et al., 2011), suggesting that the mutualist coating the seed is likely to dominate the root system, at least early in the season. In addition, we have long known of the diverse genetic variation inherit in rhizobia, and seeds are often already coated with the best genotype for the host plant. Recent studies have also revealed incredible genetic variation (particularly in growth promotion of crop species) within a single species of AM fungi under selection (Ehinger et al., 2009; Angelard et al., 2010). As a result, there is the potential to create “designer” AM fungi (Sanders, 2010), and coating seed with these AM fungi could have dramatic effects on crop yields. Additionally, different isolates of the same AM fungal species produce different aboveground herbivore defense patterns within host plants (Wooley and Paine, 2007), suggesting there is also the potential to select for AM fungi that promote particular defensive strategies within the host as well as crop yields. Belowground symbionts, however, are not the only symbionts with breeding potential. Endophytes (both fungal and bacterial) in aboveground tissues can also exhibit wide genetic variation, and there is research to suggest that viruses which insert genetic material can change the mutualistic behavior of fungal endophytes (reviewed in Bao and Roossinck, 2013). Many herbivorous insects also host symbionts, and these symbionts also have great genetic variation, upon which breeding programs could also act (Frago et al., 2012). As a result, there are significant non-plant breeding opportunities that could increase production limits and improve food security.

Here we have shown that there two steps we could take to utilize above–belowground interactions to increase production limits thereby leading to enhanced food security: intercropping and new crop and microbial breeding strategies.

## CONCLUSION

We have demonstrated above that there is potential to manage above–belowground interactions to form an important part, amongst social, cultural, political, technological, and scientific changes in assisting to Reduce the Yield Gap, and Increase Production Limits. Above–belowground interactions in themselves will not be able to exclusively combat these issues, however increased understanding of these interactions form part of a holistic solution to improving food security that incorporates tools and technologies from a diverse range of fields. There are many challenges associated with achieving food security, but managing above–belowground interactions and defensive characteristics of plants could play an important role. An added advantage of managing above–belowground interactions for increasing yields is the lack of chemical inputs and the sustainability of these approaches to agriculture. As such, managing above–belowground interactions is likely a lower cost option, especially as the cost of chemical inputs continues to rise, and is likely a more reasonable option for the world’s poorest farmers.

Managing above–belowground interactions to promote food security also raises the issue of a lack of a “best fit” solution. Similarly, many studies within this area of research have been conducted in glasshouses, and a wide range of field trials are needed to determine the real world effects of these interactions and their potential for application within agricultural systems, as laboratory results do not always translate to successes in the field. With more factors influencing the relationships, we see increased variation in results, and although studies show positive mitigation can be achieved, this is not always the case (Newsham et al., 1995; Torres-Barragán et al., 1996; Bødker et al., 2002). As noted above, variations in biotic and abiotic factors such as the species / genotype used, environmental conditions, and a range of other factors can have a strong influence on relationships and interactions, and above–belowground interactions can reduce yields as easily as improve them. Populations of beneficial organisms may already be present within agricultural systems, leading to little observed increases in practice, though scope remains to tailor and target these associations for maximum benefit to crops. Above–belowground interactions, like climate and pest distributions, vary spatially and temporally. In order to best utilize above–belowground interactions in agriculture we will need to understand how these interactions vary spatially and temporally. There are several systems in which this information is already available (such as herbivore densities), but several other systems where more information needs to be gathered. Despite the lack of information we have highlighted here several opportunities for utilizing these interactions to promote crop yields.

## Conflict of Interest Statement

The authors declare that the research was conducted in the absence of any commercial or financial relationships that could be construed as a potential conflict of interest.
